# Association between chronic obstructive pulmonary disease and 28-day mortality in patients with sepsis: a retrospective study based on the MIMIC-III database

**DOI:** 10.1186/s12890-023-02729-5

**Published:** 2023-11-09

**Authors:** Yubiao Chen, Lifei Lu, Xicong Li, Baiyun Liu, Yu Zhang, Yongxin Zheng, Yuan Zeng, Ke Wang, Yaru Pan, Xiangning Liang, Zhongji Wu, Yutian Fu, Yongbo Huang, Yimin Li

**Affiliations:** 1 Department of Critical Care Medicine, the First Affiliated Hospital of Guangzhou Medical University, Guangzhou, 510120 China; 2 Guangzhou Institute of Respiratory Health, Guangzhou, 510120 China; 3 State Key Laboratory of Respiratory Diseases, Guangzhou, 510120 China; 4 National Center for Respiratory Medicine, National Clinical Research Center for Respiratory Disease, The First Affiliated Hospital of Guangzhou Medical University, Guangzhou, 510120 China; 5 Department of Cardiology, Kunming Medical University, the 920th Hospital, Kunming, 650032 Yunnan China; 6 Hubei University of Medicine, Shiyan, Hubei 442000 China

**Keywords:** Sepsis, Chronic obstructive pulmonary disease, MIMIC-III, All-cause mortality

## Abstract

**Background:**

Sepsis is a common cause of mortality in critically ill patients, and chronic obstructive pulmonary disease (COPD) is one of the most common comorbidities in septic patients. However, the impact of COPD on patients with sepsis remained unclear. Therefore, the purpose of this study aimed to assess the effect of COPD on the prognosis of septic patients based on Medical Information Mart for Intensive Care (MIMIC-III) database.

**Methods:**

In this retrospective study based on the (MIMIC)-III database version 1.4 (v1.4), we collected clinical data and 28-day all-cause mortality from patients with sepsis in intensive care unit (ICU) and these patients met the diagnostic criteria of Sepsis 3 on ICU admission between 2008 and 2012. International Classification of Diseases (ICD-9) (4660, 490, 4910, 4911, 49120, 49121, 4918, 4919, 4920, 4928, 494, 4940, 4941, 496) was used to identified COPD. We applied Kaplan–Meier analysis to compare difference of 28-day all-cause mortality between septic patients with and without COPD. Cox proportional-hazards model was applied to explore the risk factor associated with 28-day all-cause mortality in patients with sepsis.

**Results:**

Six thousand two hundred fifty seven patients with sepsis were included in this study, including 955 (15.3%) patients with COPD and 5302 patients without COPD (84.7%). Compared with patients without COPD, patients with COPD were older (median: 73.5 [64.4, 82.0] vs 65.8 [52.9, 79.1], *P* < 0.001), had higher simplified acute physiology score II (SAPSII) (median: 40.0 [33.0, 49.0] vs 38.0 [29.0,47.0], *P* < 0.001) and greater proportion of mechanical ventilatory support (MV) (55.0% vs 48.9%, *P* = 0.001). In our study, septic patients with COPD had higher 28-day all-cause mortality (23.6% vs 16.4%, *P* < 0.001) than patients without COPD. After adjusting for covariates, the results showed that COPD was an independent risk factor for the 28-day all-cause mortality of patients with sepsis (HR 1.30, 95%CI: 1.12–1.50, *P* = 0.001).

**Conclusions:**

COPD was an independent risk factor of 28-day all-cause mortality in septic patients. Clinically, septic patients with COPD should be given additional care.

## Background

Sepsis is a life-threatening organ dysfunction caused by the dysregulated host response to infection [1]. It is one of the major health problems in the world. Although progress in medical sciences has led to a decrease in sepsis prevalence and mortality, about 20% of deaths worldwide were attributed to sepsis [2].

Chronic obstructive pulmonary disease (COPD) emerged as a critical factor contributing to the therapy and prognosis of patients with sepsis [3–5]. Approximately 6.9%-16.5% of septic patients had COPD, and COPD was one of the most prevalent chronic complications in patients with sepsis [6–13]. There existed a potential interconnection between sepsis and COPD on pathogenesis. COPD patients not only exhibited pulmonary inflammation but a state of persistent systemic inflammation. A meta-analysis of  W Gan et al. [14] demonstrated that compared with patients without COPD, patients with COPD had elevated levels of circulating leucocytes, fibrinogen, C-reactive protein (CRP) and tumour necrosis factor-α (TNF-α). In addition to systemic inflammation, patients with stable COPD exhibited a prothrombotic state. An observational study revealed that patients with stable COPD showed elevated levels of crucial coagulation factors (FII, FV, FVIII and FX), while exhibiting reduced levels of coagulation inhibitors (protein S and antithrombin) [15]. Oxidative stress played a significant role in the pathogenesis of COPD. In another study, COPD patients presented higher level of 8-isoprostane (a marker of ongoing oxidative stress) in sputum compared with control subject [16]. Given that dysregulated immune response, coagulation disorders and excessive production of oxidants were inherent features of sepsis [17, 18], systemic inflammatory [14], prothrombotic state [15, 19] and oxidative stress [20] in patients with COPD may contribute to the progressions of sepsis. Likewise, the occurrence of sepsis can also serve as a trigger for acute exacerbations of COPD. Chen et al. [21] found that the onset of sepsis was associated with a poor long-term prognosis characterized by increased risks of severe exacerbations, mortality, pneumonia, and serious pneumonia in patients with COPD. However, the relationship between COPD and progressions of sepsis remained controversial. One retrospective study included 22,354 sepsis patients found that COPD was associated with 60-day mortality [6]. On the contrary, several multi-center studies had reported that COPD was not associated with in-hospital mortality in septic patients [7–9, 22–24]. Furthermore, the relationship between COPD and short-term mortality in septic patients remained unclear.

Therefore, this retrospective study aimed to explore the impact of COPD on 28-day all-cause mortality in patients with sepsis through the Medical Information Mart for Intensive Care (MIMIC)-III database version 1.4 (v1.4) spinning from 2018–2012.

## Methods

### Database

Data from MIMIC-III v1.4 which is a large, single-center, publicly available critical care database were acquired for conducting this study. This database was composed of adult patients (aged 16 years or above) admitted to critical care units in the Beth Israel Deaconess Medical Center (BIDMC, Boston Massachusetts, USA) between 2001 and 2012. The database consisted of records involving demographics, vital signs, laboratory tests, fluid balance and vital status; documents International Classification of Diseases and Ninth Revision (ICD-9) codes; documents hourly physiologic data from bedside monitors confirmed by intensive care unit (ICU) nurses; and stores written evaluations of radiologic films by specialists covering in the corresponding time period. One author (Yubiao Chen) completed the CITI Data or Specimens Only Research couse, and was approved to gain access to the database and took responsibility for data extraction (certification number 40416369). Since this study was conducted using an anonymous public database that satisfies the protocol of the review board, the requirement of ethical consent is not necessary.

### Study population

We focused on septic patients defined by sepsis-3 criteria [1]; Briefly, patients with documented or suspected infection and an acute change in total Sequential Organ Failure Assessment (SOFA) [25] score of ≥ 2 points were defined as sepsis. COPD was identified using International Classification of Diseases (ICD-9) codes (4660, 490, 4910, 4911, 49,120,49,121, 4918, 4919, 4920, 4928, 494, 4940, 4941, 496) [26]. We removed patients admitted to ICU before 2008 as the method was described previously [27], because antibiotic prescriptions have only been recorded since 2003, and groups admitted between 2008 and 2012 were easily identified in the database.

**Fig. 1:**
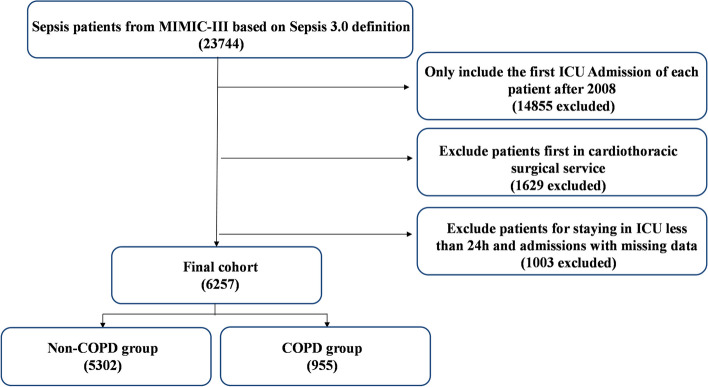
Study cohort. Illustration of exclusion and inclusion criteria as utilized to select the final cohort of 6257 patients

Thirty eight thousand six hundred five MIMIC-III subjects in admissions were included in our study, and 23,744 were identified as sepsis. Then 14,855 secondary (or more) ICU admission were removed, and we included only 8889 first ICU admissions for patients from 2008 – 2012. 1629 patients who were first in cardiothoracic surgical service were excluded since their post-operative physiologic derangements do not translate to the same mortality risk as the other ICU patients. After excluding 1003 patients with less than 24 h of ICU stay and admissions with missing data, we finally included 6257 septic patients in our cohort, of which 955 (15.3%) were known to have COPD (Fig. 1).

### Data extraction

Structured Query Language (SQL), PostgreSQL tools (version 9.6) and STATA version 17.0 were applied for data extraction and management. Data about age, sex, ethnicity, comorbidities, the first laboratory parameters after ICU admission, two scoring systems containing the SOFA score and simplified acute physiology score II (SAPSII) [28], mechanical ventilation (MV) when ICU admission, the length of hospital and ICU stay and date of dead of patients were extracted directly or calculated. The comorbidities identified by ICD-9 code included atrial fibrillation (AFIB), coronary artery disease (CAD), congestive heart failure (CHF), diabetes, malignancy, chronic renal disease, liver disease, stroke and the laboratory parameters included hemoglobin, platelet counts, white blood cell counts, the percentage of lymphocyte and neutrophil, neutrophil-to-lymphocyte ratio (NLR), PH, partial pressure of oxygen (PO_2_), partial pressure of carbon dioxide (PCO_2_), bicarbonate, partial-thromboplastin time (PTT), prothrombin time (PT), glucose, urea nitrogen, creatinine, lactate, creatine kinase, MB Isoenzyme (CK-MB), creatine kinase, alanine transaminase (ALT), aspartate transaminase (AST), alkaline phosphatase (ALP) and albumin. Outcomes in our study contained 7-, 14-, 21- and 28-day all-cause mortality, mortality in the ICU, in-hospital mortality and length of ICU and hospital stay.

### Statistical analysis

Baseline characteristics and the laboratory parameters of all patients were stratified by the comorbidity of COPD. After test of normality distribution by Kolmogorov–Smirnov test, continuous data were presented as median and interquartile range (IQR) and significant differences between two group was tested by Wilcoxon rank-sum test. Categorical data were reported as frequencies and percentages and were compared using chi-square test. To determine the association between the comorbidity of COPD with 28-day all-cause mortality, we presented Kaplan–Meier curve and analyzed the result using log-rank tests. Furthermore, the Cox proportional-hazards model was used to analyze the independent effects of COPD on 28-day all-cause mortality. Forward stepwise method was applied for variables selection for Cox proportional-hazards models. Sensitive analyses stratified by sex and MV were performed to explore the association between COPD and 28-day all-cause mortality. Except that Kaplan–Meier approach for survival analysis was performed by R software, the data were analyzed with the statistical package IBM SPSS Statistics software (SPSS) (v16.0; IBM, Armonk, NY). A two-tailed *P* < 0.05 was considered as statistically significant.

## Results

### Population characteristics

Baseline characteristics and laboratory parameters of the study population were shown in Tables 1 and 2. Patients with COPD were significantly older (median: 73.5 [64.4, 82.0] vs 65.8 [52.9, 79.1], *P* < 0.001), had higher SAPSII scores (median: 40.0 [33.0, 49.0] vs 38.0 [29.0, 47.0], *P* < 0.001), and lager percentage of receiving mechanical ventilation at admission (55.0% vs 48.9%, P = 0.001) than patients without COPD. Septic patients with COPD were more likely to have atrial fibrillation (38.8% vs 26.8%, P < 0.001), coronary artery disease (33.4% vs 23.8% *P* < 0.001) and congestive heart failure (44.1% vs 25.7%, *P* < 0.001). There was no significant difference in proportion of diabetes (31.3% vs 29.8%, *P* = 0.337) and malignancy (22.6% vs 21.3%, *P* = 0.366) between two groups (Table 1). Compared with patients without COPD, patients with COPD exhibited a higher percentage of neutrophil (median: 84.0 [75.8, 89.6] vs 82.5 [74.0, 88.3], *P* < 0.001) and a lower percentage of lymphocyte (median: 9.0 [5.0, 14.6] vs 9.8 [5.8, 15.9], *P* < 0.001), and an elevated neutrophil to lymphocyte ratio (median: 9.0 [5.2, 16.8] vs 8.3 [4.7, 14.6], *P* = 0.002). Besides, septic patients with COPD had higher platelet counts (median: 208.0 [149.0, 288.3] vs 196.0 [113.0, 273.0], *P* < 0.001), PTT (median: 31.1 [26.6, 37.9] vs 30.2 [26.3, 36.9], *P* = 0.036), as well as PCO2 (median: 45.0[38.0, 55.0] vs 40.0 [34.0, 45.0], *P* < 0.001), and a lower PH (median: 7.37 [7.30, 7.43] vs 7.39 [7.34, 7.44], *P* < 0.001) and PO2 (median: 92.0 [69.0, 135.0] vs 113.0 [80.0, 171.0], *P* < 0.001) than septic patients without COPD (Table 2).

**Table 1:** Characteristics of patients with sepsis at the admission to ICU according to the presence of COPD

Independent variable	Total (*N* = 6257)	Non-COPD (*N* = 5302)	COPD (*N* = 955)	*P*
Age, Years	67.3 (54.3, 79.8)	65.8 (52.9, 79.1)	73.5 (64.4, 82.0)	** < 0.001**
Sex, n (%)				0.114
Male	3514 (56.2)	3000 (56.6)	514 (53.8)	
Female	2743 (43.8)	2302 (43.4)	441 (46.2)	
Body mass index (kg/m2)	27.1 (23.6, 31.9)	27.2 (23.6, 31.6)	27.0 (23.3, 33.4)	0.123
Ethnicity, n (%)				** < 0.001**
White	4549 (72.7)	3799 (71.7)	750 (78.5)	
Black	626 (10.0)	544 (10.3)	82 (8.6)	
Hispanic	232 (3.7)	214 (4.0)	18 (1.9)	
Other	850 (13.6)	745 (14.1)	105 (11.0)	
Comorbidities, n (%)				
AFIB	1793 (28.7)	1422 (26.8)	371 (38.8)	** < 0.001**
CAD	1582 (25.3)	1263 (23.8)	319 (33.4)	** < 0.001**
CHF	1782 (28.5)	1361 (25.7)	421 (44.1)	** < 0.001**
Diabetes	1877 (30.0)	1578 (29.8)	299 (31.3)	0.337
Malignancy	1346 (21.5)	1130 (21.3)	216 (22.6)	0.366
Renal	1427 (22.8)	1165 (22.0)	262 (27.4)	** < 0.001**
Liver	558 (8.9)	495 (9.3)	63 (6.6)	**0.006**
Stroke	577 (9.2)	510 (9.6)	67 (7.0)	**0.001**
Scoring systems, median				
SOFA	5.0 (3.0, 7.0)	5.0 (3.0, 7.0)	5.0 (3.0, 7.0)	0.240
SAPSII	38.0 (30.0, 48.0)	38.0 (29.0,47.0)	40.0 (33.0, 49.0)	** < 0.001**
Intervention				
MV, n (%)	3119 (49.8)	2594 (48.9)	525 (55.0)	**0.001**

*Abbreviations:* *AFIB* Atrial fibrillation, *CAD* Coronary artery disease, *CHF* Congestive heart failure, *COPD* Chronic obstructive pulmonary disease, *MV* Mechanical ventilation, *SOFA* Sequential organ failure assessment, *SAPSII* Simplified acute physiology score II. Bold text indicates *P* < 0.05

**Table 2:** Laboratory parameters of patients with sepsis at the admission to ICU according to the presence of COPD

Laboratory parameters, median	Total (*N* = 6257)	Non-COPD (*N* = 5302)	COPD (*N* = 955)	*P*
Hemoglobin, g/dL	10.3 (9.1, 11.7)	10.3 (9.1, 11.8)	10.3 (9.1, 11.6)	0.496
Platelet counts, 10^3^/μL	197.0 (133.0, 275.0)	196.0 (113.0, 273.0)	208.0 (149.0, 288.3)	** < 0.001**
White blood cell, 10^3^/μL	10.3 (7.4, 14.0)	10.3 (7.4, 14.0)	10.5 (7.4, 14.2)	0.368
Neutrophil, %	82.8 (74.0, 88.6)	82.5 (74.0, 88.3)	84.0 (75.8, 89.6)	** < 0.001**
Lymphocyte, %	9.7 (5.7, 15.7)	9.8 (5.8, 15.9)	9.0 (5.0, 14.6)	** < 0.001**
NLR	8.4 (4.8, 14.9)	8.3 (4.7, 14.6)	9.0 (5.2, 16.8)	**0.002**
Eosinophil, %	0.4 (0.1, 1.4)	0.5 (0.1, 1.4)	0.4 (0.1, 1.2)	0.121
PH	7.39 (7.33 7.44)	7.39 (7.34, 7.44)	7.37 (7.30, 7.43)	** < 0.001**
PaO_2,_ mm Hg	109.0 (77.0, 166.0)	113.0 (80.0, 171.0)	92.0 (69.0, 135.0)	** < 0.001**
PaCO_2,_ mm Hg	40.0 (35.0, 47.0)	40.0 (34.0, 45.0)	45.0 (38.0, 55.0)	** < 0.001**
Bicarbonate, mEq/L	24.0 (21.0, 27.0)	24.0 (21.0, 27.0)	26.0 (23.0, 30.0)	** < 0.001**
INR	1.2 (1.1, 1.5)	1.2 (1.1, 1.5)	1.2 (1.1, 1.5)	0.541
PT, s	14.2 (12.9, 16.5)	14.3 (12.9, 16.6)	14.0 (12.8, 16.1)	0.052
PTT, s	30.3 (26.4, 37.1)	30.2 (26.3, 36.9)	31.1 (26.6, 37.9)	**0.036**
Glucose, mg/dL	123.0 (102.0, 153.0)	122.0 (102.0, 152.0)	124.0 (103.0, 157.0)	0.180
Creatinine, mg/dL	1.0 (0.7, 1.6))	1.0 (0.7, 1.7)	1.0 (0.7, 1.6)	0.465
Urea nitrogen, mg/dL	22.0 (14.0, 37.0)	21.0 (14.0, 36.0)	25.0 (16.0, 40.0)	** < 0.001**
Lactate, mmol/L	1.6 (1.1, 2.4)	1.6 (1.2, 2.4)	1.4 (1.0, 2.0)	** < 0.001**
CK-MB, ng/mL	5.0 (3.0, 9.0)	5.0 (3.0, 9.0)	5.0 (3.0, 8.0)	0.611
Creatine kinase, IU/L	113.0 (52.0, 321.0)	121.0 (55.0, 346.0)	90.0 (42.5, 210.0)	** < 0.001**
ALT, IU/L	28.0 (17.0, 59.0)	29.0 (17.5, 61.0)	24.0 (15.0, 52.5)	** < 0.001**
AST, IU/L	37.0 (23.0, 75.0)	38.0 (23.0, 77.5)	31.0 (20.8, 64.2)	** < 0.001**
ALP, IU/L	86.0 (61.0, 129.8)	87.0 (62.0, 133.0)	83.0 (60.5, 115.0)	**0.021**
Albumin, g/dL	3.1 (2.6, 3.6)	3.1 (2.6, 3.6)	3.1 (2.7, 3.5)	0.964

*Abbreviations:* *ALP* Alkaline phosphatase, *AST* Aspartate transaminase, *ALT* Alanine transaminase, *CK-MB* Creatine kinase, MB Isoenzyme, *INR* International Normalized Ratio, *NLR* Neutrophil-to-Lymphocyte Ratio, *PT* Prothrombin Time, *PTT* Partial-thromboplastin time. Bold text indicates *P* < 0.05

### Outcomes of septic patients with and without COPD

Compared with septic patients without COPD, septic patients with COPD had higher mortality at any visit periods (especially 28-day all-cause mortality [23.6% vs 16.4%, *P* < 0.001]), higher mortality in ICU (13.5% vs 8.9%, *P* < 0.001), in-hospital mortality (17.1% vs 12.3%, *P* < 0.001) and longer length of ICU stay (3.1 days vs 2.9 days, *P* = 0.005) (Table 3).

**Table 3:** Outcomes of patients with sepsis according to the presence of COPD

Outcome	Total (*N* = 6257)	Non-COPD (*N* = 5302)	COPD (*N* = 955)	*P*
Mortality, n (%)				
7-day	536 (8.6)	437 (8.2)	99 (10.4)	**0.031**
14-day	797 (12.7)	647 (12.2)	150 (15.7)	**0.003**
21-day	975 (15.6)	778 (14.7)	197 (20.6)	** < 0.001**
28-day	1097 (17.5)	872 (16.4)	225 (23.6)	** < 0.001**
ICU	600 (9.6)	471 (8.9)	129 (13.5)	** < 0.001**
Hospital	813 (13.0)	650 (12.3)	163 (17.1)	** < 0.001**
Length of stay, median (IQR)				
Hospital, day	7.2 (4.6, 12.1)	7.2 (4.6, 12.1)	7.1 (4.7, 12.1)	0.489
ICU, day	2.9 (1.8, 5.8)	2.9 (1.8, 5.6)	3.1 (1.9, 6.4)	**0.005**

*Abbreviations:* ICU, Intensive care unit. Bold text indicates *P* < 0.05

### Risk factors associated with 28-day all-cause mortality in septic patients

**Fig. 2:**
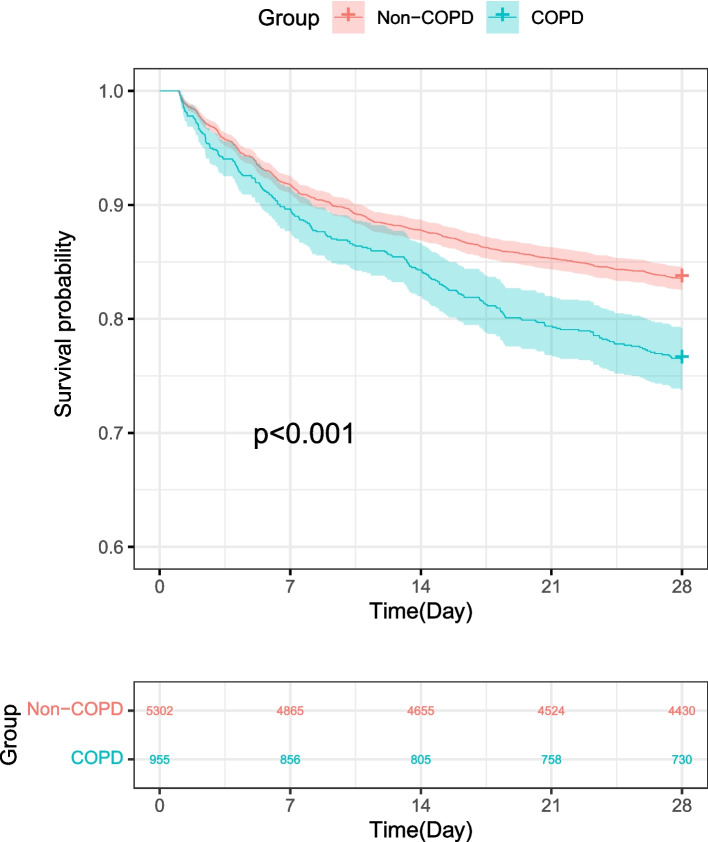
Kaplan–Meier analysis of the probability of survival of patients with sepsis according to the presence of COPD

**Fig. 3:**
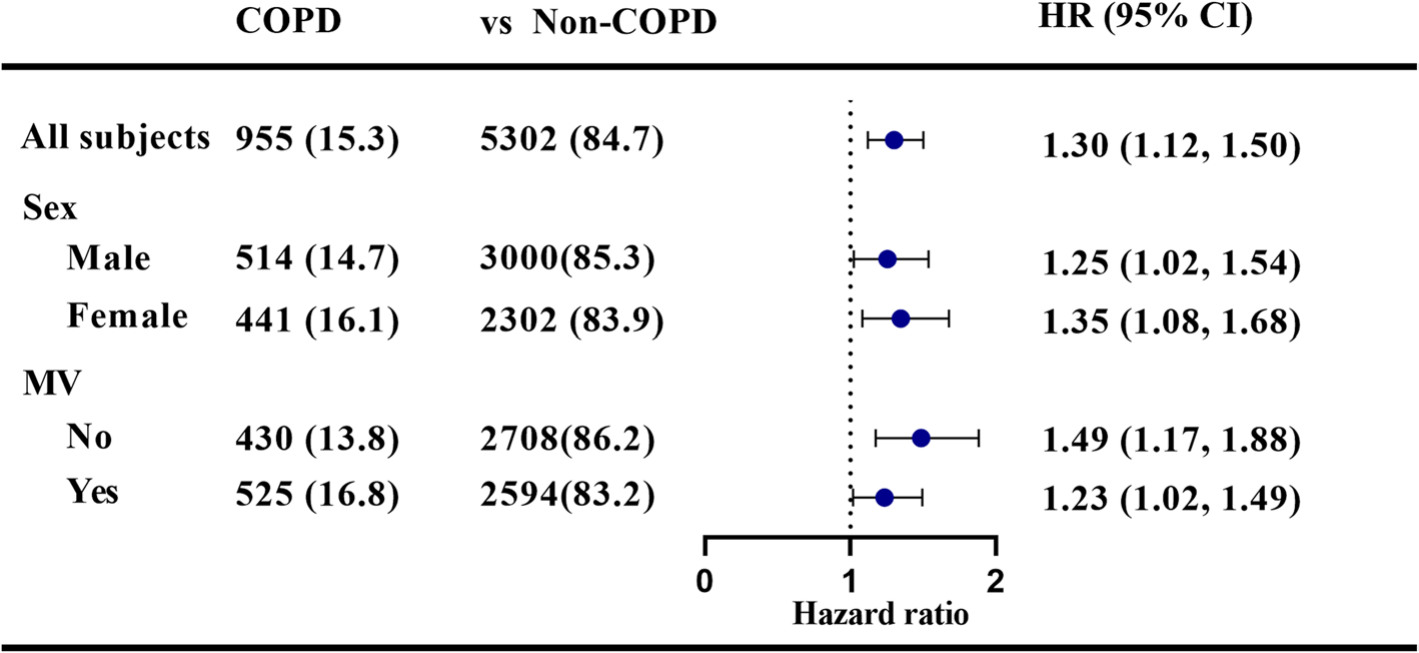
Sensitive analysis of the associations between COPD and 28-day mortality among sepsis patients. Abbreviations: *MV* Mechanical ventilation

Kaplan–Meier curves in Fig. 2 showed that septic patients with COPD had worse short-term survival rates. Furthermore, we performed univariate analysis to explore risk factors associated with 28-day all-cause mortality in septic patients and found age, ethnicity, comorbidities (COPD, AFIB, CHF, Malignancy, Renal, Liver, Stroke), scoring systems (SOFA, SAPSII) and mechanical ventilation were associated with 28-day all-cause mortality in septic patients, and similar result was found in the multivariate analysis. As a result, the comorbidity of COPD remained a significant risk factor of 28-day all-cause mortality (HR 1.30, 95%CI: 1.12–1.50, *P* = 0.001) (Table 4). Besides, sensitive analyses stratified by sex and MV were employed to evaluate the association between COPD and 28-day all-cause mortality in septic patients, and the results in each subgroup were in accordance with those in overall patients (Fig. 3).

**Table 4:** Cox proportional hazard models exploring the association between COPD and 28-day mortality among septic patients

	Univariate model		Multivariate model	
	Hazard ratio (95% CI)	*P*	Hazard ratio (95% CI)	*P*
COPD	1.48 (1.28, 1.71)	**< 0.001**	1.30 (1.12,.1.50)	**0.001**
Age (Years)	1.026 (1.02, 1.03)	**< 0.001**	1.01 (1.007, 1.02)	** < 0.001**
Sex (Male)	0.98 (0.87, 1.10)	0.687	Not selected	-
Ethnicity				
White	Reference		Reference	-
Black	0.77 (0.62, 0.97)	**0.021**	0.86 (0.69, 1.08)	0.197
Hispanic	0.80 (0.57, 1.13)	0.210	1.13 (0.79, 1.60)	0.508
Other	1.30 (1.10, 1.52)	**0.002**	1.25 (1.06, 1.47)	**0.007**
Comorbidities				
AFIB	1.72 (1.52, 1.94)	**< 0.001**	1.20 (1.06, 1.37)	**0.006**
CAD	0.99 (0.87, 1.14)	0.933	0.83 (0.72, 0.95)	**0.009**
CHF	1.36 (1.20, 1.54)	** < 0.001**	Not selected	
Diabetes	0.99 (0.87, 1.13)	0.909	Not selected	
Malignancy	1.87 (1.64, 2.11)	** < 0.001**	1.58 (1.38, 1.80)	** < 0.001**
Renal	1.32 (1.15,1.50)	**< 0.001**	Not selected	
Liver	1.52 (1.27, 1.82)	**< 0.001**	1.50 (1.24, 1.82)	** < 0.001**
Stroke	1.62 (1.36, 1.93)	**< 0.001**	1.80 (1.51, 2.14)	** < 0.001**
Scoring systems, median				
SOFA	1.18 (1.16, 1.20)	**< 0.001**	1.05 (1.02,1.07)	** < 0.001**
SAPSII	1.054 (1.05,1.06)	**< 0.001**	1.04 (1.03,1.05)	** < 0.001**
Intervention				
MV	1.39 (1.23,1.56)	**< 0.001**	Not selected	

Multivariate model was adjusted for age, ethnicity, comorbidities (COPD, AFIB, CAD, Malignancy, Liver, Stroke), and scoring systems (SOFA, SAPSII). Abbreviations: *AFIB* Atrial fibrillation, *CAD* Coronary artery disease, *CHF* Congestive heart failure, *COPD* Chronic obstructive pulmonary disease, *MV* Mechanical ventilation, *SOFA* Sequential organ failure assessment, *SAPSII* Simplified acute physiology score II. Bold text indicates *P* < 0.05

## Discussion

This study has revealed two significant findings. Our results showed that septic patients with COPD were older, had more comorbidities, higher SAPSII score, and higher proportion of mechanical ventilation than septic patients without COPD. Secondly, we found that COPD was an independent risk factor for 28-day all-cause mortality in patients with sepsis.

This study showed that 15.3% COPD existed in patients with sepsis, and this result was consisted with the previous studies that the proportion of COPD accounted for 6.9%-16.5% in patients with sepsis [6–13]. A global audit of ICU data reported that there were 454 (15.2%) septic patients with COPD [9]. A demographic study from Sweden showed that in critically ill patients, COPD accounted for 10.5% of 22,354 septic patients [6]. The multi-center, large sample study reported that 12.1% of septic patients were with COPD [13]. These studies indicated that COPD was one of the most common comorbidities of patients with sepsis.

We found that patients with COPD were older and had more comorbidities, higher SAPSII score, as well as higher proportion of mechanical ventilation support than those without COPD in patients with sepsis. Moreover, septic patients with COPD had higher NLR, lower PO_2_, lower PH, and higher PCO_2_, due to systemic inflammation [14], chronic hypoxemia [29] and hypercapnia [30]. Taken together, the results revealed that septic patients with COPD had worse status than those without COPD when admitted in ICU.

We further explored the risk factors of 28-day all-cause mortality in patients with sepsis. The result of cox proportional-hazards model showed that COPD was an independent risk factor for 28-day all-cause mortality in patients with sepsis. Our study was consistent with the study of Björn Ahlström et al. [6] that COPD was the risk factor for 60-day mortality in septic patients. However, some studies had contradictory results. Multi-center studies from China reported that COPD was not associated with sepsis-related in-hospital mortality in patients with sepsis [7, 8]. Furthermore, a study from multiple ICUs around the world also showed that COPD was not associated with in-hospital mortality in septic patients [9]. The contradictory results may be due to the differences in the definition of sepsis and the admission time of patients with sepsis in the ICU. In this study, the criteria of sepsis were based on the sepsis-3, which greatly assessed the magnitude of the problem of patients with sepsis [1]. Besides, this study only included patients diagnosed as sepsis within 24 h before and after admission to the ICU to avoid the interference of different sepsis infection patterns caused by acquired infections in the ICU.

We tried to figure out the reason why septic patients with COPD had poorer prognosis. Firstly, patients with COPD were older than those without COPD, and older age may be associated with the poor prognosis of these patients [10, 31, 32]. Secondly, cardiovascular diseases were associated with poor prognosis in patients with COPD [33], and also was associated with poor prognosis in patients with sepsis [34, 35]. In this study, septic patients with COPD had higher proportion of cardiovascular system diseases. Systemic inflammation, hypoxia, and prothrombotic status in COPD patients may increase the risk of cardiovascular events in septic patients [36, 37]. In addition, the results of laboratory tests showed that compared with septic patients without COPD, the platelet counts and NLR were higher in sepsis patients with COPD. NLR was an indicator of systemic inflammation based on complete blood count values, and associated with the onset and development of inflammatory diseases [38, 39]. A recent meta-analysis study also showed that higher NLR was associated with poor prognosis in sepsis [40]. Delayed neutrophil apoptosis, increased release of immature neutrophils and lymphocyte apoptosis can lead to significant increase in circulating neutrophils and lymphopenia in septic patients, and resulted in sepsis progression through enhancing neutrophil-mediated killing and dysregulated innate immune responses [41]. Therefore, except the common risk factors, poorer prognosis of septic patients with COPD may also be related to dysregulated innate immune responses.

This study also existed some limitations. Firstly, COPD was defined by ICD-9 codes [26] rather than based on the spirometry (post-bronchodilator forced expiratory volume in one second/forced vital capacity ratio < 0.70) [42], resulting in the overdiagnosis of COPD [43]. Secondly, since forced expiratory volume in one second and smoking history were important risk factors for patients with COPD [42], due to lack of the two indicators in the MIMIC-III database, the interference caused by these two factors cannot be ruled out.

## Conclusions

Our study provided evidence that COPD was an independent risk factor for 28-day all-cause mortality in septic patients. These findings highlighted the importance of providing specialized care for septic patients with COPD. However, it is crucial to recognize that the association between COPD and higher mortality in septic patients is still hypothetical and requires further investigation through appropriately designed studies.

## Data Availability

Available upon request. Corresponding to Drs. Yongbo Huang (yongbo2046@163.com) or Yimin Li (dryiminli@vip.163.com).

## Abbreviations

AFIB: Atrial fibrillation
CAD: Coronary artery disease
CHF: Congestive heart failure
COPD: Chronic obstructive pulmonary disease
NLR: Neutrophil-to-Lymphocyte Ratio
PO_2_: Partial pressure of oxygen
PCO_2_: Partial pressure of carbon dioxide
PTT: Partial-thromboplastin time
PT: Prothrombin time
CK-MB: Creatine Kinase, MB Isoenzyme
ALT: Alanine transaminase
AST: Aspartate transaminase
ALP: Alkaline phosphatase
